# An unusual neurological manifestation of granulomatosis with polyangiitis: A case report and literature review

**DOI:** 10.1002/ccr3.2765

**Published:** 2020-02-29

**Authors:** Nikhil N. Tarte, Ronald Ceruti, Vasudev Tati

**Affiliations:** ^1^ Baton Rouge General Internal Medicine Residency Program Baton Rouge General Medical Center Baton Rouge LA USA; ^2^ The Baton Rouge Clinic Baton Rouge LA USA

**Keywords:** aspirin, central nervous system, dysphagia, granulomatosis with polyangiitis, hypercoagulable state, stroke, vasculitis, Wegener's granulomatosis

## Abstract

Ischemic stroke is an incredibly rare manifestation of granulomatosis with polyangiitis. It is important for the clinician to be aware of this unusual complication so that efforts can be made to reduce the risk of this event.

## Graphical abstract

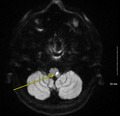

## 1 INTRODUCTION

Granulomatosis with polyangiitis (GPA), or Wegener's granulomatosis, is a necrotizing vasculitis affecting small‐ to medium‐sized vessels. It usually involves the nose, sinuses, lungs, and kidneys. Neurological symptoms such as peripheral neuropathy are actually quite common. However, central nervous system (CNS) involvement, on the other hand, is rather rare. Here, we discuss the case of a patient with GPA who presented with dysphagia, who was thought to have an infectious etiology that was easily curable, but turned out to be something more sinister.

## 2 CASE REPORT

The patient is a 47‐year‐old Caucasian man presenting to the emergency department with dysphagia. He states that about an hour after his dinner the previous night, he had production of thick mucus with inability to swallow. He slept well that night, but the next morning, he reported that he had difficulty swallowing both pills and liquids. He also admitted to some mild hoarseness.

The patient reports that he had a Wegener's flare about three months prior which involved paresthesias of the lower extremities, sinusitis, and leukocytoclastic vasculitis. He was treated with pulse dose steroids, rituximab, and prednisone. His prednisone had been titrated down to 20 mg twice a day at the time of presentation. He also stated that he had an endoscopy done 1 month prior due to dental erosions, where he was found to have evidence of esophageal candidiasis.

He denied any other symptoms including fever/chills, cough, hemoptysis, shortness of breath, sore throat, heartburn, chest pain, abdominal pain, hematuria, or upper and lower extremity weakness/numbness.

His past medical history included hyperlipidemia and granulomatosis with polyangiitis that was diagnosed 13 years ago. At that time, he presented with a sinus flare and was put into remission by Cytoxan. His GPA manifestations have included sinusitis, saddle‐nose deformity, peripheral neuropathy, and skin lesions. He has never had pulmonary or renal involvement. He drinks alcohol socially. He denies smoking or illicit drug use. Surgical history and family history are noncontributory.

**Figure 1:**
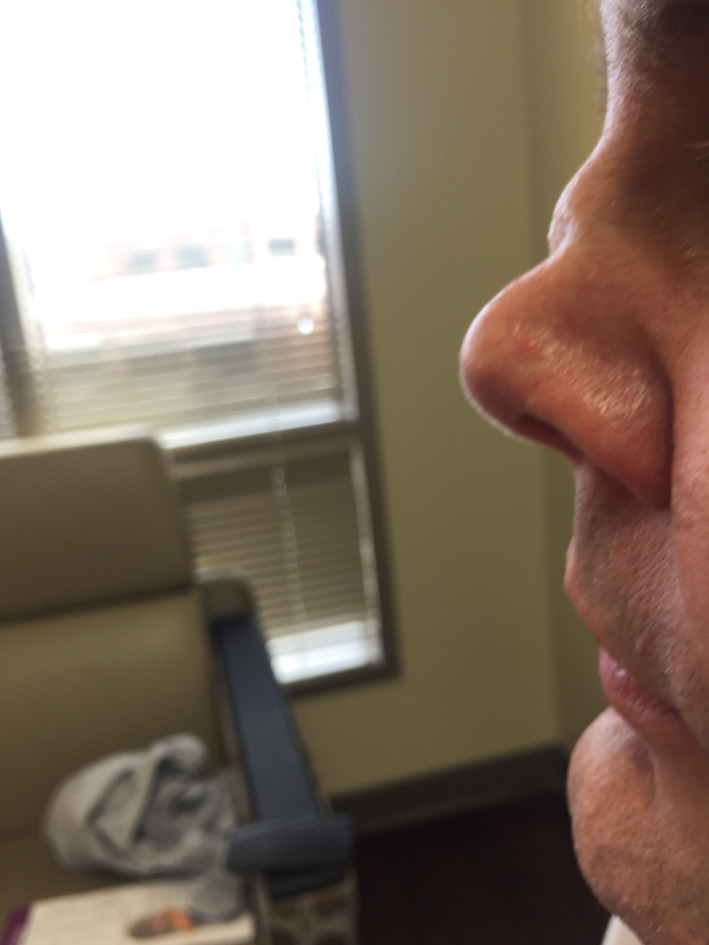
Saddle‐nose deformity

**Figure 2:**
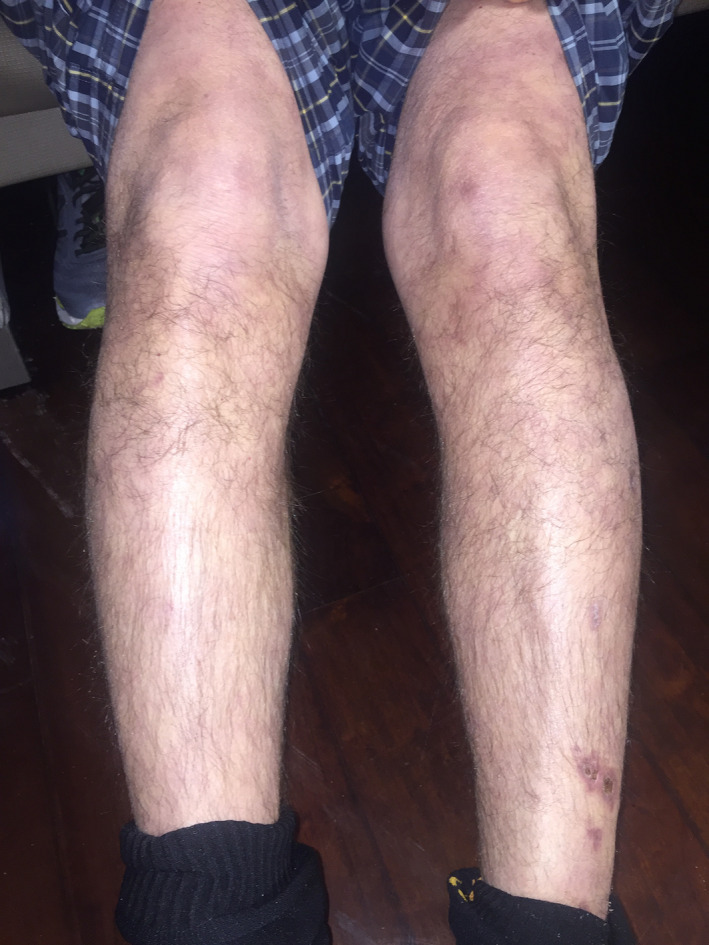
Livedo reticularis

On arrival to the emergency room, patient was tachycardic at 128 beats/min and was slightly hypertensive at 144/86 mm Hg. On physical examination, he was a middle‐age Caucasian man in no acute distress. He did have a rather Cushingoid appearance. Pupils were equally round and reactive to light. Oropharyngeal examination revealed oral thrush on his tongue without evidence of erythema or ulcers. Nasal turbinates were of normal appearance. Saddle‐nose deformity was present (Figure 1). There was no lymphadenopathy. Cardiovascular examination did not reveal any abnormal heart sounds. Lung sounds were clear. Abdomen was soft and nontender. Upon neurological examination, cranial nerves II‐XII were intact. Motor and sensory examinations were intact. He had no dysdiadochokinesis or dysmetria. His gait was normal. Skin examination revealed livedo reticularis on his lower extremities with some healed crusted lesions (Figure 2).

**Figure 3:**
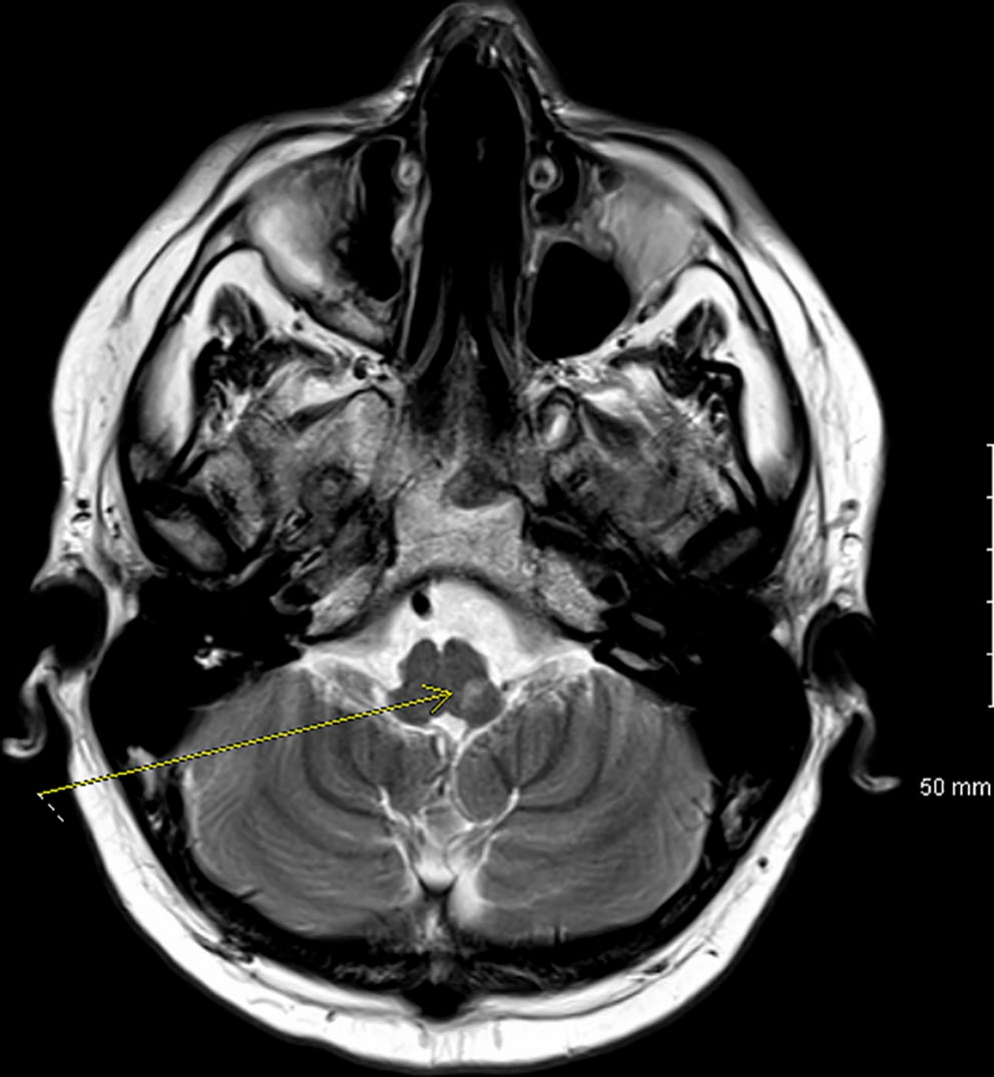
Axial T2 image showing left medullary stroke (yellow arrow)

**Figure 4:**
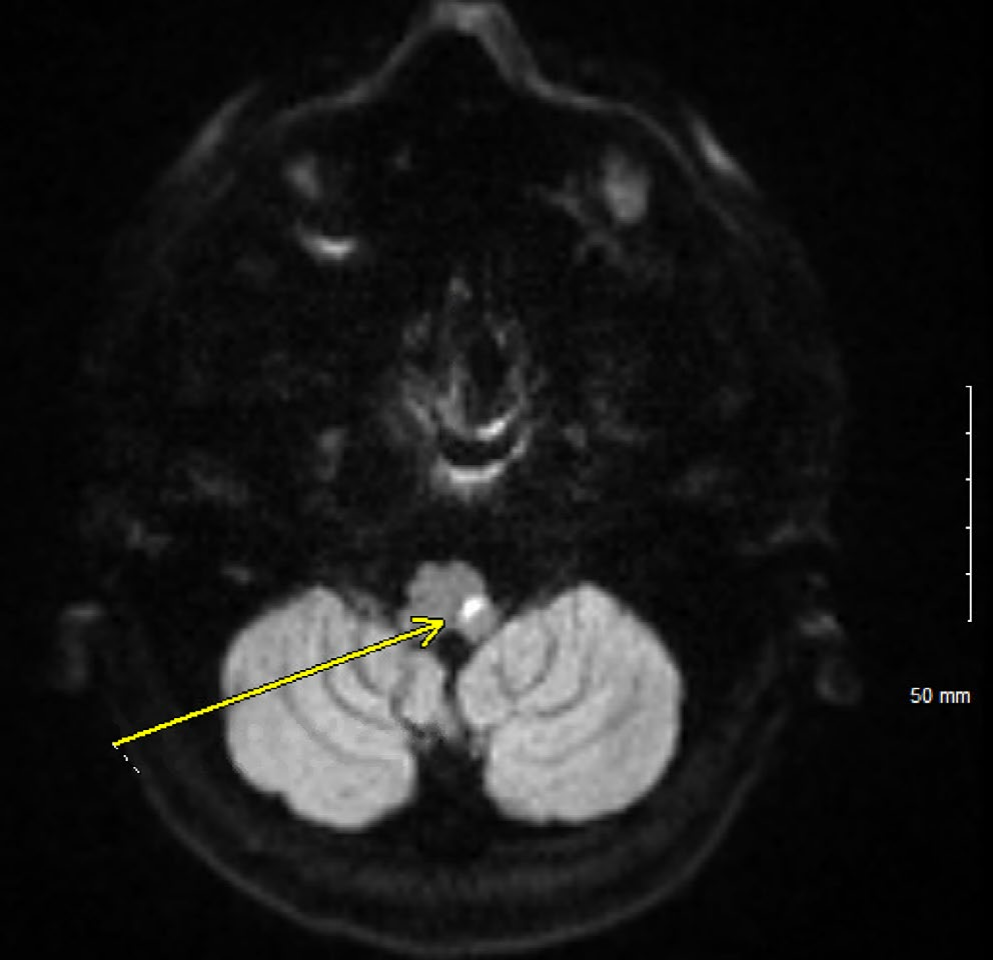
Increased DWI signal is seen in the medulla indicating acute infarction (yellow arrow)

**Figure 5:**
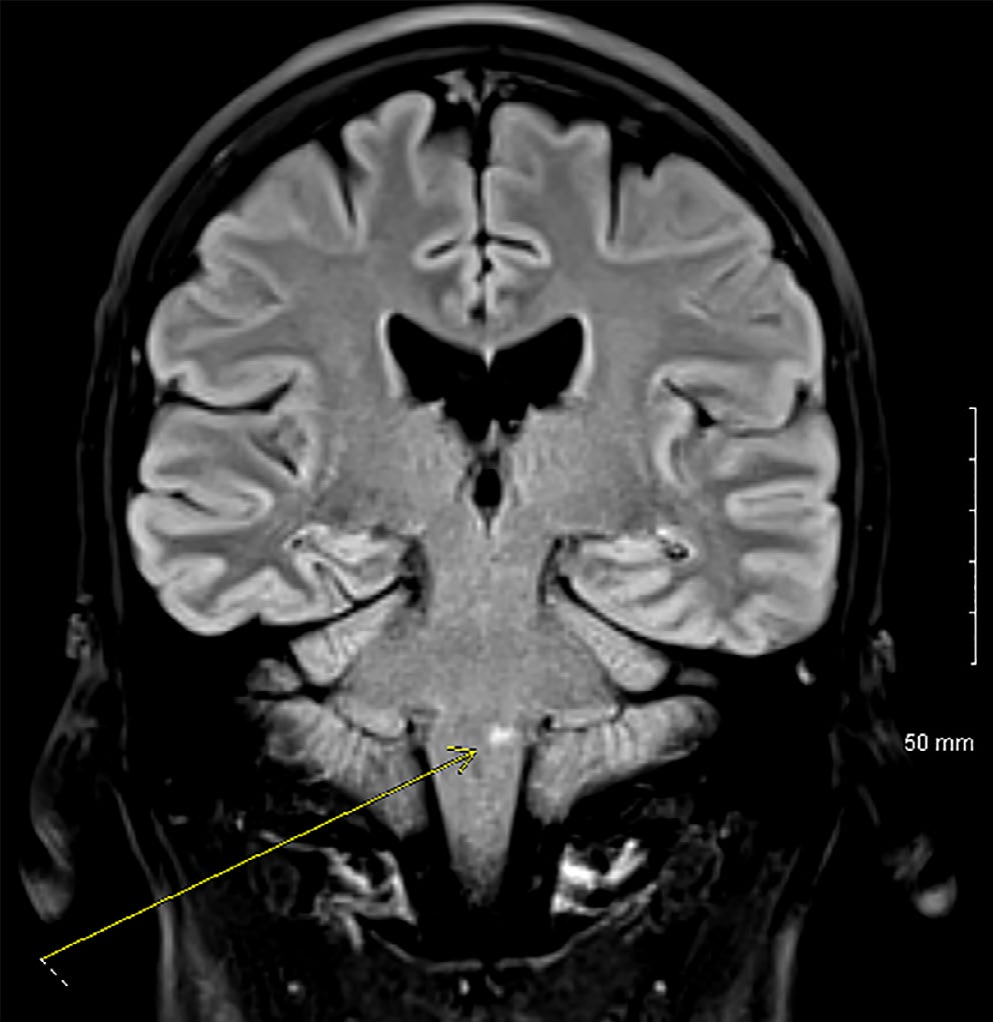
Coronal image showing T2 FLAIR hyperintensity of the left medullary region (yellow arrow)

**Figure 6:**
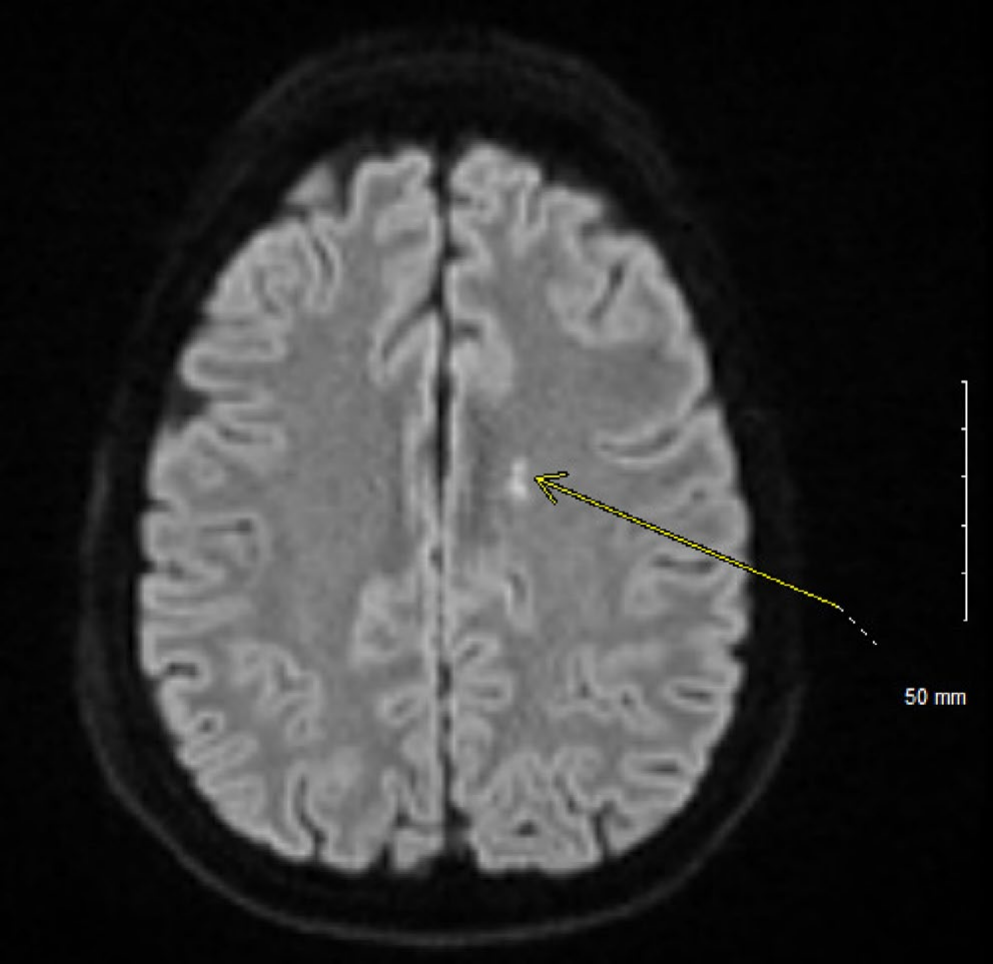
There are two foci of restricted diffusion within the left centrum semiovale characteristic of acute infarction (yellow arrow)

In the emergency department, electrocardiogram (EKG) revealed sinus tachycardia. Laboratories were largely unremarkable except a slight leukocytosis of 11.4 K/µL and albumin of 3.0 g/dL. Screening for mononucleosis was negative, and rapid Group A Strep screen was also negative. An esophagram was obtained which showed an irregular shaggy appearance of the esophagus. Due to the fact that he was on steroids, he was started on fluconazole for presumed esophageal candidiasis and admitted to the hospital. A pharyngogram was obtained which showed severe oropharyngeal dysphagia; however, these findings were not consistent with esophageal Candidiasis. Other etiologies for his dysphagia had to be entertained, including possible cranial nerve involvement of his Wegener's. A bedside laryngoscopy was performed which showed a slightly rotated larynx and abnormal vocal cord function. Neurologist was consulted who noted that the patient had an absent gag reflex. Computed tomography (CT) of the neck with IV contrast was gotten which was unremarkable Therefore, magnetic resonance imaging (MRI) of the head was ordered which revealed an acute left medullary infarct (Figures 3, 4, and 5) as well as two acute lacunar infarcts involving the left centrum semiovale (Figure 6). Lipid panel was abnormal with a total cholesterol of 239 mg/dL, LDL of 223 mg/dL, HLD of 33 mg/dL, and triglycerides of 290 mg/dL. His glycated hemoglobin (HbA1c) was 5.8%. Echocardiogram demonstrated normal ejection fraction, left ventricular hypertrophy, and normal left atrial chamber without thrombus. Due to his relatively young age and the fact that he had only one risk factor for stroke, we could not attribute his stroke to only hyperlipidemia. Other causes of stroke had to be evaluated including vasculitis or hypercoagulability. Computed tomography angiography (CTA) of the head was negative for vasculopathy. Anticardiolipin, lupus anticoagulant, and beta‐2 glycoprotein were all negative. C‐ANCA was positive at 1:40, and anti‐PR3 was positive at 52.9 U/mL. P‐ANCA was negative. Both erythrocyte sedimentation rate (ESR) and C‐reactive protein (CRP) were elevated at 45 mm/h and 2.2 mg/dL, respectively. A lumbar puncture was done which was negative for infection and only showed a mildly elevated CSF protein of 77 mg/dL.

The patient was started on aspirin in addition to high intensity statin which he was already on. Azathioprine was added by his rheumatologist, and his steroid taper was continued. Over the course of his hospital stay, he slowly had improvement of his dysphagia and hoarseness. A repeat pharyngogram still showed significant dysphagia, and the patient had to undergo percutaneous endoscopic gastrostomy tube (PEG) placement with tube feeds. Patient was discharged in stable condition with instructions to follow‐up with his rheumatologist.

## 3 DISCUSSION

Granulomatosis with polyangiitis is a necrotizing granulomatous inflammatory disease affecting small‐ to medium‐sized vessels. The disease usually manifests in the upper and lower airways as well as in the kidneys.^1^ Common symptoms include fever, fatigue, weight loss, myalgias, purulent nasal discharge, epistaxis, nasal mucosal ulceration, otitis media, hemoptysis, and hematuria. Saddle‐nose deformity is a common physical examination finding. Laboratory markers are generally nonspecific. There may be anemia, thrombocytosis, elevated ESR and CRP, elevated creatinine, and active urinary sediment. GPA is also highly associated with c‐ANCA and anti‐PR3 which is high in sensitivity and specificity. Diagnosis is clinicopathologic and involves confirmation with biopsy of the lung, kidney, or sinus. Treatment is with corticosteroids and cyclophosphamide.^2^ Other agents with less toxicity have been used including rituximab and azathioprine.^3^

In this case, our patient presented with a rather unusual complication, a stroke. Neurological manifestations can happen in GPA and are not uncommon. In a cohort of 128 patients, half the patients (50%) had neurological symptoms. About 43.8% had involvement of the peripheral nervous system (sensorimotor polyneuropathy or mononeuritis multiplex).^4^ However, CNS manifestations are rather uncommon in GPA. In the same cohort, only 7% had involvement of the CNS. Also, six of the same 128 patients (4.7%) had involvement of the cranial nerves including the optic, oculomotor, trigeminal, and facial nerves.^4^ Of those patients with CNS involvement, symptoms include headache, sensory and motor impairment, vestibular syndrome, mood disturbances, and hearing loss.^5^ Stroke, on the other hand, remained an even more uncommon manifestation of the CNS. In a retrospective study of 35 patients with GPA who had CNS involvement, de Luna G, et al found that 9 out of the 35 patients (25.7%) had evidence of ischemic stroke on MRI.^5^ Stroke has been found as both a presenting symptom and as a complication of GPA. Emamikhah et al documented a case of a middle‐age female patient who presented with a lateral medullary stroke who was interestingly found to have underlying GPA.^6^ Solik and Fick reported a case of patient who was admitted to the hospital for a GPA flare and then subsequently developed a debilitating ischemic stroke as a complication from her GPA.^3^

This unusual case prompts the question, does GPA lead to an increased risk of ischemic stroke? According to a cohort study conducted in Canada, the authors found that those with GPA had an increased incidence of ischemic stroke, 8.9 per 1000 person‐years compared with 4.3 per 1000 person‐years in healthy controls; however, the results were not significant.^7^ Although our patient had elevated lipids at the time of diagnosis, he had no other risk factors for stroke. Being relatively young and only having one risk factor, something else had to have increased his predisposition to having an ischemic event. Review of the literature suggests that there is an association between ANCA‐associated vasculitis (AAV) and venous thromboembolic events (VTE), about 7 per 100 person‐years compared with 0.15‐0.31 in the general population.^8^ Hilhorst M, et al investigated hypercoagulability in patients with ANCA‐associated vasculitis who were in remission using the endogenous thrombin potential (ETP), an indicator of the degree of hypercoagulability. He found that among 31 patients who had AAV, 27 of those patients had an elevated ETP, with the mean average significantly higher than that of the healthy controls. Factor VIII levels were elevated on average as well, suggesting that endothelial cell dysfunction could be one mechanism leading to a prothrombotic state.^9^ While a hypercoagulable state is well implicated in the pathophysiology of VTE, there is also evidence suggesting a role in predisposing to cerebral ischemia of arterial origin, especially in young patients. In an observational study of 100 patients under the age of 55 diagnosed with ischemic stroke or transient ischemic attack, around 46% of the patients had either an inherited or acquired hypercoagulable disorder.^10^

One important point to note is that this patient presented with a medullary infarct as well as two *lacunar* infarcts on imaging. Lacunar infarcts result from occlusion of penetrating arteries supplying the deep structures of the brain.^11^ While hypertension constitutes the main risk factor and cause of lacunar strokes, <5% of cases are caused by various etiologies including hematological disorders and vasculitis.^12^ Therefore, one such etiology to consider in this patient is vasculitis of the CNS, as this has been implicated as a cause of stroke in those with GPA.^5^ However, this mechanism is less likely given that the CTA in this patient did not show any vascular abnormality. A biopsy of the brain can be useful to confirm the diagnose of vasculitis if suspicion is high; however, this was not attempted in this patient. As a result, hypercoagulability remains the most plausible explanation for this patient's multiple strokes. In one study, investigators found increased levels of fibrinogen, von Willebrand factor, and thrombomodulin in elderly patients with lacunar infarcts compared with healthy subjects, leading the authors to conclude that hypercoagulability and endothelial cell damage are associated with lacunar strokes, especially in patients with multiple strokes.^13^

Armed with this knowledge, is there any intervention that can be used to reduce the risk of this complication such as the use of antiplatelet agents? In a retrospective study of 48 patients with Takayasu's arteritis, it was found that those who suffered from arterial ischemic events were less likely to be taking antiplatelet agents (14%) than those who did not have ischemic events (82%), *P* < .0001. Antiplatelet therapy was associated with a decreased risk of ischemic events (hazard ratio = 0.055, *P* = .011). 14 In another retrospective study, Nesher et al found that those patients with giant cell arteritis (GCA) who were on low‐dose aspirin were less likely to develop cerebral vascular events (CVAs) and acute vision loss than those who were not on aspirin (*P* < .01).^15^

## 4 CONCLUSION

We conclude that GPA may lead to an increased risk of ischemic stroke by inducing a hypercoagulable state, possibly via endothelial cell dysfunction. Even though the above studies demonstrated a benefit of using aspirin in patient with Takayasu's or GCA, no study has been done which investigates the employment of primary prevention of stroke in patients diagnosed with AAV. More research needs to be done to elucidate the benefits and the risks of antiplatelet agents in these patients who are at increased risk of thromboembolism. It is important for the clinician to be aware of this unusual complication, so that more effort can be made to reduce modifiable risk factors for stroke in those with GPA.

## CONFLICT OF INTEREST

None declared.

## AUTHOR CONTRIBUTIONS

NT: wrote the manuscript and did literature review. RC: he was the rheumatologist on the case and looked over the manuscript and made edits and recommendations. VT: he was the attending physician on the case who looked over the whole process.
